# A toroidal SAW gyroscope with focused IDTs for sensitivity enhancement

**DOI:** 10.1038/s41378-024-00658-9

**Published:** 2024-03-15

**Authors:** Lu Tian, Haitao Zhao, Qiang Shen, Honglong Chang

**Affiliations:** Ministry of Education Key Laboratory of Micro and Nano Systems for Aerospace, School of Mechanical Engineering, Northwestern Polytechnical University, Xi’an, 710072 China

**Keywords:** Sensors, NEMS

## Abstract

A surface acoustic wave (SAW) gyroscope measures the rate of rotational angular velocity by exploiting a phenomenon known as the SAW gyroscope effect. Such a gyroscope is a great candidate for application in harsh environments because of the simplification of the suspension vibration mechanism necessary for traditional microelectromechanical system (MEMS) gyroscopes. Here, for the first time, we propose a novel toroidal standing-wave-mode SAW gyroscope using focused interdigitated transducers (FIDTs). Unlike traditional SAW gyroscopes that use linear IDTs to generate surface acoustic waves, which cause beam deflection and result in energy dissipation, this study uses FIDTs to concentrate the SAW energy based on structural features, resulting in better focusing performance and increased SAW amplitude. The experimental results reveal that the sensitivity of the structure is 1.51 µV/(°/s), and the bias instability is 0.77°/s, which are improved by an order of magnitude compared to those of a traditional SAW gyroscope. Thus, the FIDT component can enhance the performance of the SAW gyroscope, demonstrating its superiority for angular velocity measurements. This work provides new insights into improving the sensitivity and performance of SAW gyroscopes.

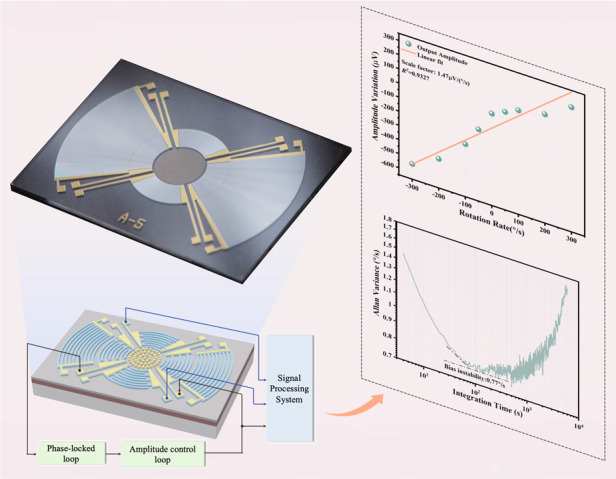

## Introduction

As a mechanical wave propagating along a solid surface^1,2^, a surface acoustic wave (SAW) is the basis for a variety of applications, including oscillators^3–5^, delay lines^6,7^, and filters^8–12^. Compared to gigahertz (GHz) electromagnetic waves, SAWs travel approximately five orders of magnitude slower and remain confined to the surface of the medium. These characteristics enable easy acquisition and processing of SAWs, facilitating signal transmission in a compact structure with minimal crosstalk between devices^13^. Therefore, SAW sensors using SAW devices as the sensing element, which convert external physical information into SAWs of various velocities or frequencies, have been extensively studied^14–18^.

The SAW gyroscope, which is an embodiment of SAW technology, harnesses the intrinsic characteristics of standing wave and progressive wave modes to measure the rotational angular velocity, grounded in the principle of the gyroscope effect^19–23^. The SAW gyroscope has a simplified structure of the floating movable components of the traditional microelectromechanical system (MEMS) gyroscope, a greatly improved capability to resist shock vibration of the gyroscope, and a reduced production cost, making it an excellent candidate for application in harsh environments. Nevertheless, owing to the mechanism characteristics of the surface acoustic wave sensing model, the weak Coriolis force and gyroscopic effect hinder attainment of high-precision physical measurements, curtailing the practical application of the SAW gyroscope.

The key components of a SAW gyroscope include linear interdigitated transducers (IDTs), metallic pillars, and reflectors. Using the SAW gyroscope effect, the rotational angular velocity of the device can be converted into a voltage or frequency for angular velocity signal detection. To enhance the gyroscope effect, Woods^24^, Haekwan Oh^25,26^, and Wen Wang et al.^27–29^ placed metallic pillars along the SAW propagation path and increased the Coriolis force by increasing the vibration mass of a particle, thus enhancing the gyroscope sensitivity. However, an increase in the size of the metallic pillars inevitably leads to attenuation of the transmitted acoustic signal and increased difficulty of the manufacturing process^30^. Furthermore, traditional linear IDTs cause surface acoustic wave diffraction^31,32^, refraction^33,34^, and beam steering^35–37^ during the transmission of SAWs, which causes energy dissipation, degrades the Coriolis force, and severely affects the sensitivity. In focused IDTs, the design of the electrodes is changed to transform the straight-line structure into an arc-shaped structure, leading to a better focusing performance, maximizing the acoustic force gradient, and increasing the amplitude of the SAW^38,39^. In contrast to conventional IDTs, FIDTs concentrate the SAW energy at the center of the IDT with a higher intensity, a high beam width compression ratio and a small localized area while a lower driving power is applied to achieve the same vibration amplitude.

Based on this, for the first time, we propose a toroidal SAW gyroscope using focused IDTs (FIDTs) and experimentally evaluate the performance of the gyroscope. The results prove that the FIDT structure significantly enhances the sensitivity of the SAW gyroscope. In this paper, the principle and design of our SAW gyroscope are first elucidated, the influence of structural parameters on the gyroscope is analyzed, and an expression for the sensitivity of the gyroscope is obtained. After theoretical analysis, the device can be produced on a wafer by the MEMS lift-off method. Subsequently, the static and dynamic performances of the gyroscope are evaluated through experiments. Through experimental analysis, it is proven that the FIDTs can improve the performance of the gyroscope. In “Discussion” and “Conclusion”, the factors influencing the performance of the proposed toroidal SAW gyroscope are discussed, which provides ideas for future research, and the article is summarized with a performance comparison between this work and previously reported studies.

## Design and results

### Device design and simulation

**Fig. 1:**
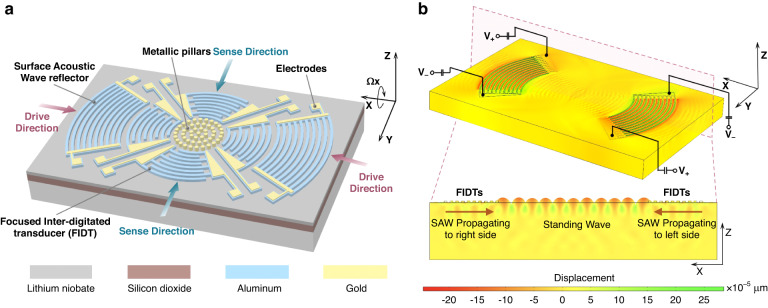
Working principle of the new toroidal SAW gyroscope. **a** The proposed gyroscope consists of FIDTs, circular arc reflectors, metallic pillars, and electrodes. The structure takes advantage of the Coriolis effect so that the amplitude of the secondary SAW generated by the Coriolis force is proportional to the angular velocity of the gyroscope, which is detected by a sensing FIDT. **b** The same alternating voltages are applied to the FIDTs in the driving direction to excite oppositely traveling SAWs, and the two SAWs are superimposed to form a standing wave

As demonstrated in Fig. 1a, a surface acoustic wave gyroscope based on the piezoelectric effect mainly consists of FIDTs, circular arc reflectors, metallic pillars, and electrodes sputtered on the surface of the device. The FIDTs are periodically spaced metal electrodes placed on a piezoelectric material that provide efficient transduction between alternating electric fields and acoustic waves. In a SAW gyroscope, the IDT structures are divided into two parts, the driving electrode and the sensing electrode, both of which are composed of a group of IDTs and are placed in a plane with an orthogonal arrangement. By applying an alternating voltage with the same operating frequency to the FIDTs on both sides along the driving direction, the standing wave mode can be generated to serve as a stable reference vibration for the gyroscope. To augment the efficiency of standing wave generation, pairs of reflectors are designed on both sides of the driving FIDTs. The reflectors in the driving direction are in the form of a short circuit, and the interdigital finger structure of the reflectors is interfaced with metal electrodes to achieve the short-circuit effect. Due to the electrical characteristics being similar to those of IDTs, the reflector structure results in an induced potential of the SAW of zero, thus enhancing the reflection efficiency. The metallic pillars are arranged in the center of the structure and move periodically following the standing wave, which is similar to the function of the suspended proof mass of a MEMS gyroscope. When out-of-plane rotation *Ω*_*x*_ is applied to the device (along the X axis), the metallic pillars located at the antinodes of the standing wave generate the secondary SAW attributable to the Coriolis force, and its propagation direction is perpendicular to that of the standing wave. The external angular rate can be calculated by detecting the signal of the secondary SAW utilizing the sensing FIDTs in the sensing direction.

According to the working principle of a SAW gyroscope, a standing wave is generated by an alternating voltage applied to the driving electrode. The standing wave mode is beneficial for strengthening the amplitude of a particle located at an antinode along the Z direction, leading to a high vibration velocity. To better reveal the formation principle of standing waves, a finite element model of the SAW gyroscope driving structure is established based on the commercial software COMSOL. The 128° Y-cut thin-film-LN-on-insulator (LNOI) model is simulated by using the hybrid physical field of solid-state mechanics and the piezoelectric effect. The Euler formula is used to correct the characteristic values of the piezoelectric material, such as the elastic stiffness and piezoelectric coefficient, to obtain more accurate simulation results. Furthermore, low reflection boundary conditions are applied to all surfaces at the bottom and sides of the simulated device to minimize the reflection effect caused by elastic waves at these boundary surfaces. Figure 1b demonstrates the evolution stages of spatially localized intersecting SAWs in the full 3D simulation of SAWs emanating from opposing sets of IDTs on a piezoelectric LiNbO_3_ substrate. The maximum displacement amplitudes at the complete intersection for the left and right sides are shown in the cross-sectional view. The particle distributed at an antinode of the standing wave has the periodically largest amplitude, which guides us to arrange the metallic pillars at the largest amplitudes to obtain the maximal inertia force. Notably, using a standing wave to generate a stable vibration velocity is key for an SAW gyroscope to simplify the suspension vibration mechanism necessary for a traditional MEMS gyroscope to evolve it into a simple planar structure.

**Fig. 2:**
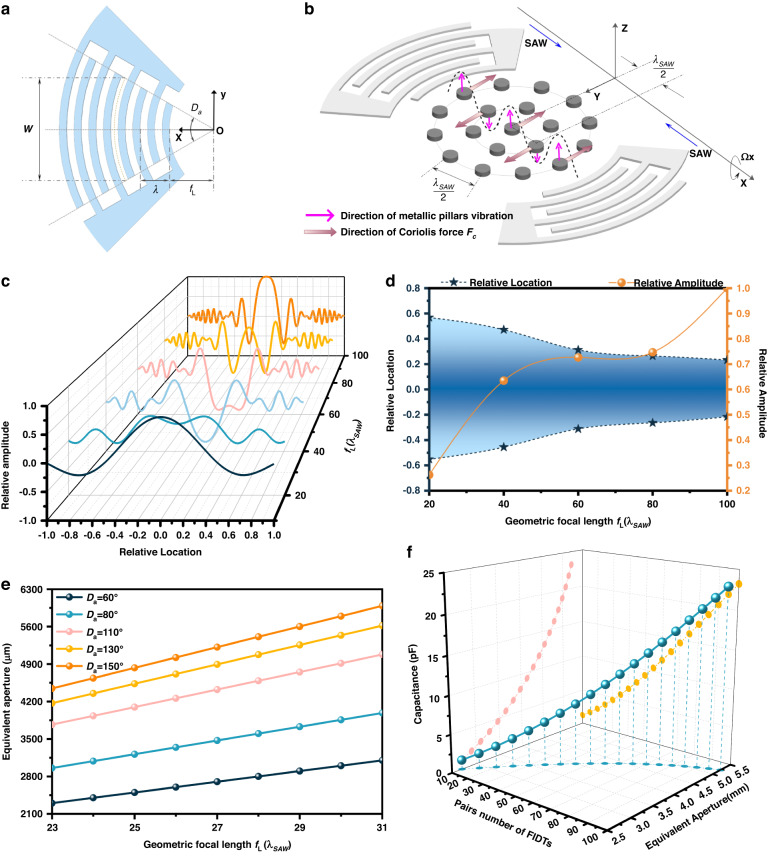
FIDT characteristics. **a** Schematic diagram of the FIDT structural parameters. **b** The metallic pillars located at the antinodes of the standing wave can increase the strength of the Coriolis force, which causes a secondary SAW. **c** FIDT response characteristics for various geometric focal lengths. **d** The geometric focal length has an effect on both the active area and the vibration amplitude of the SAW. The left axis shows the relative transmission range depending on the geometric focal length. The right axis shows the relative amplitude curves for different geometric focal lengths. **e** Equivalent aperture for different degrees of arc *D*_a_. **f** The capacitance of the FIDTs is affected by the number of pairs and the equivalent aperture

To improve the performance of the SAW gyroscope, this paper designs a shaped driving structure different from the traditional linear interdigitated transducer (LIDT), that is, the focused interdigitated transducer (FIDT), as depicted in Fig. 2a. The FIDT is composed of interdigitated fingers based on concentric wave surfaces, and the key structural parameters include the FIDT arc angle (*D*_*a*_), geometric focal length (*f*_*L*_), equivalent aperture (*W*) and acoustic wavelength (*λ*_*SAW*_). By specially designing the electrode structure in the SAW device, the traditional linear IDTs are converted into circular focus FIDTs that localize the SAW energy around the center of the circle. The focused acoustic energy maximizes the acoustic force gradient and produces surface acoustic waves with a larger amplitude. Due to their structural characteristics, FIDTs can suppress beam deflection and concentrate more SAW energy in the center of the interdigitated transducers while maintaining a higher strength, a larger beam-width compression ratio and a confined area of action. According to the theoretical equation of the Coriolis force^40^, the strength depends on the mass of the vibrating particle, the vibration velocity of the vibrating particle and the external rotational angular velocity. To increase the Coriolis force, many studies^41,42^ have adopted various schemes, such as increasing the proof mass and increasing the amplitude of the driving mode. In this work, by properly placing metallic pillars along the SAW propagation path, the Coriolis force can be effectively improved, thereby enhancing the gyroscopic effect. As shown in Fig. 2b, the metallic pillars are arranged at the antinodes of the standing wave, and the spacing is an integer multiple of half the wavelength to maximize the vibration velocity. Moreover, the direction in which the Coriolis force acts on the metallic pillars changes with time, and the Coriolis force in the same direction ensures codirectional enhancement.

Another way to improve the strength of the Coriolis force is to increase the velocity of particle vibration. In this work, utilizing the structural characteristics of FIDTs, the energy of the SAW is converged, which is beneficial for increasing the vibration velocity of the particle. To better elaborate on this principle, by selecting a single particle, the vibration displacement of the particle can be obtained using the characteristics of acoustic wave transmission in the piezoelectric medium, which is expressed as^43–45^:

1 $${u}_{i}={i}_{0}^{{\prime} }\,\cos (\omega t-\frac{2\pi }{{\lambda }_{SAW}}X)(i=x,z)$$

where *u*_*i*_ is the particle SAW displacement along the *i*th direction; ${i}_{0}^{{\prime} }$ is the amplitude affected by temperature along the *i*th direction; *ω* is the angular velocity, which can be expressed as $\omega =2\pi {v}_{{SAW}}^{{\prime} }/{\lambda }_{{SAW}}=2\pi {v}_{{SAW}}(1-\alpha \Delta T)/{\lambda }_{{SAW}}={\omega }_{{SAW}}(1-\alpha \Delta T)$; *ω*_*SAW*_ is the angular velocity at the resonant frequency; *λ*_*SAW*_ is the acoustic wavelength; *X* is the displacement between the focal point and acoustic aperture, which can be expressed as $X={f}_{L}{W}^{2}/{\lambda }_{{SAW}}\left(1+\gamma \right)$; *f*_*L*_ is the geometric focal length; *W* is the equivalent aperture of the central finger of FIDTs, which can be expressed as^39,46^ $W=2r\sin \left({D}_{a}/2\right)$; *r* is the distance between the focal point and central finger; *D*_*a*_ is the FIDT arc angle; and *γ* is the anisotropy constant.

According to Eq. (1), we can obtain the SAW amplitude distribution diagram under various geometric focal lengths, as shown in Fig. 2c. With increasing geometric focal length, the SAW generated by the FIDTs is localized toward the geometric center, and the relative amplitude increases. However, a large geometric focal length affects the active area and amplitude of the transmitted SAW and subsequently impacts the metallic pillars in the center of the device. Hence, it is necessary to establish the tradeoff between the vibration amplitude and the active area of the SAW. Figure 2d shows the relationship between the geometric focal length, the relative active area, and the relative amplitude of the SAW. The results indicate an increase in the relative amplitude of the SAW with increasing geometric focal distance at the cost of a smaller relative active area. When the geometric focal length distance is set to below 20 times the wavelength, the relative amplitude of the surface acoustic wave will be very small, resulting in a decrease in the overall performance of the device. If this distance exceeds 60 times the wavelength, this leads to a narrow active area, which affects the Coriolis effect and leads to an increase in the size of the device. As a compromise between a large amplitude of the SAW and a greater active area of metallic pillars, the geometric focal length of the FIDTs is set to 27 times the wavelength in this design.

The standing-wave-mode SAW generated by the FIDTs in the driving direction helps the metallic pillars vibrate. When external rotational angular velocity *Ω*_*x*_ exists and the device rotates along the X-axis, the metallic pillars generate Coriolis force *F*_*c*_ along the Y-axis, which can be expressed as follows:

2 $${F}_{c}=-2{M}_{p}{\varOmega }_{x}\times {\nu }_{p}=4{M}_{p}{\varOmega }_{x}{z}_{0}^{{\prime} }\omega \,\cos \left(\frac{2\pi }{{\lambda }_{SAW}}X\right)\sin (\omega t)$$

where *M*_*p*_ is the total mass of the metallic pillars and ${z}_{0}^{{\prime} }$ is the amplitude affected by temperature alone in the z direction.

The mechanical sensitivity of the SAW gyroscope affected by temperature is expressed as the scale factor (*SF*), which can be obtained as follows (see supplementary section 1):

3 $$SF=\frac{{U}_{m}}{{\varOmega }_{x}}=\frac{4{M}_{p}d{z}_{0}^{{\prime} }{\omega }_{SAW}(1-\alpha \Delta T)}{{C}_{e}}$$

where *U*_*m*_ is the output voltage amplitude; *d* is the piezoelectric constant; *ω*_*SAW*_ is the angular velocity at the resonant frequency; *α* is the temperature coefficient; ∆*T* is the temperature variation; and *C*_*e*_ is the FIDT capacitance, which is related to the number of pairs and equivalent aperture of the FIDTs.

The influence of the structural parameters on the FIDT capacitance is shown in Fig. 2e, f. The degree of FIDT arc has an influence on the equivalent aperture, and as the degree of arc increases, the equivalent aperture correspondingly increases. Various equivalent apertures not only influence the range of the SAW generated by FIDTs but also affect the capacitance of FIDTs. Figure 2f demonstrates the interactive relationship between the number of pairs of FIDTs, equivalent aperture and capacitance. When the number of pairs of FIDTs increases from 20 to 100 and the equivalent aperture varies from 2.5 to 5, the capacitance monotonically increases. To enhance the sensitivity of the SAW gyroscope, we need to reduce the number of pairs of FIDTs and the equivalent aperture as much as possible to reduce the capacitance of the FIDTs.

### Fabrication process and measurement approach

After the device structure is designed, a 128° Y cut thin-film-LN-on-insulator (LNOI) wafer is employed for micromechanical fabrication of the SAW device. Compared with other tangential directions, the 128° Y cut LNOI has a higher electromechanical coupling coefficient, which is conducive to reducing the dissipation during energy conversion and improving the sensitivity of the SAW gyroscope. However, due to the “soft and brittle” characteristics of lithium niobate, it is easily damaged or even fractured during fabrication, which brings serious challenges to the etching process. To overcome the above challenges, we use the lift-off method to manufacture the core structures, such as interdigitated transducers and metallic pillars.

**Fig. 3:**
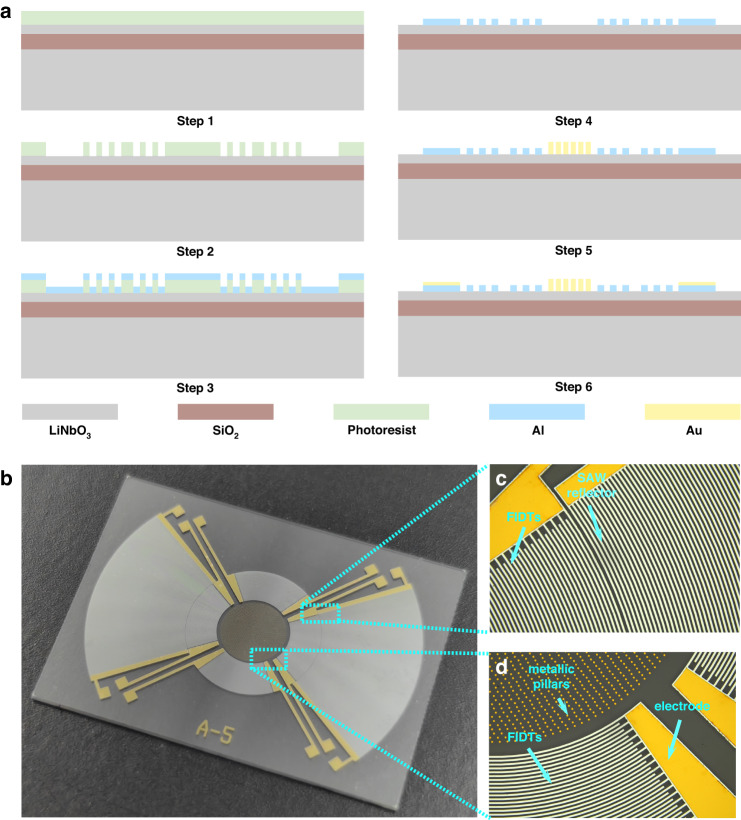
Fabrication process. **a** A lift-off process is used to manufacture the required structures. Micrograph of **b** the SAW gyroscope. **c** Zoomed-in image of the FIDTs and SAW reflectors. **d** Zoomed-in view of the metallic pillars, FIDTs, and electrodes

The detailed fabrication steps for the SAW gyroscope used in this study are shown in Fig. 3a. First, a positive photoresist (AZ4620) is selected for spin coating on the device layer. Then, a photolithography system is used for alignment and exposure to transfer the pattern onto the photoresist surface, and the photoresist is dissolved in the developing solution for 150 s. Subsequently, the FIDTs and SAW reflectors are defined by lifting off 100 nm of evaporated Al with a 9 µm photoresist layer. The same fabrication process is then repeated to deposit Au to form metal pillars with a thickness of 900 nm. Finally, using magnetron sputtering, an Au electrode with a thickness of 120 nm is defined. Figure 3b displays a micrograph of the SAW gyroscope, with the FIDTs, SAW reflectors, and metallic pillars depicted in Fig. 3c, d. The main geometric parameters of the toroidal SAW gyroscope are shown in Table 1.

**Table 1:** Key geometric parameters of the toroidal SAW gyroscope

Parameters	Value
FIDT finger period	50 μm
FIDT pairs number	23
Degree of arc	70°
Geometric focal length	2700 μm
Equivalent aperture	4531 μm
Thickness of FIDTs	100 nm
Thickness of metallic pillars	900 nm
Thickness of thin film LN	500 nm
Thickness of silicon dioxide	1.5 μm
Thickness of LN substrate	500 μm

**Fig. 4:**
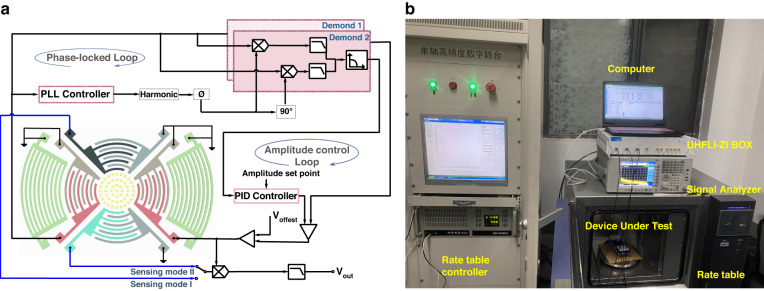
Principle of the experiment. **a** A closed-loop test scheme is used to realize phase-locked control and amplitude-stabilized control to guarantee stable vibration of the gyroscope. **b** Measurement setup

Upon completion of the fabrication of the toroidal SAW gyroscope, the open-loop test can be used to preliminarily evaluate the response characteristics of the sensor. In this experiment, the gyroscope is statically set on a high-precision rate table. The 200 mV sweep signal generated by a vector network analyzer (KEYSIGHT E5061B) is input into the driving port and the sensing port through a capacitor. The amplitude signals generated by the SAW gyroscope in the driving mode and sensing mode are amplified by a transimpedance amplifier, and then, the amplitude‒frequency characteristic curve of the gyroscope can be observed on the vector network analyzer. A schematic diagram of the closed-loop test is shown in Fig. 4a. The driving mode of the gyroscope is connected to the input and output ports of a UNFLI-ZI BOX, and the phase-locked loop locks to the resonant frequency of the driving mode. The excitation signal of the driving mode is output by Demodulator 1, where the phase shift is set to maximize the amplitude of the oscillation. Moreover, the demodulator output driving mode amplitude of the Demodulator 2 with zero phase shift serves as an input to the proportion integration differentiation (PID) controller, and the PID controller is used to compare the driving mode oscillation amplitude and regulate it to a given setpoint to realize stable amplitude control, which serves as a stable reference vibration source for the gyroscope.

The experimental setup is shown in Fig. 4b. The sensor chip is bonded on a printed circuit board (PCB) with pins jointed to an external PCB and covered by a resin lid. The device is connected to the rate table through an acrylic plate. Through the small A type (SMA) connector on the PCB, the signal can be transmitted to an external instrument for subsequent measurement. The rate table controller is used to provide the required rotational velocity to the toroidal SAW gyroscope.

## Results

**Fig. 5:**
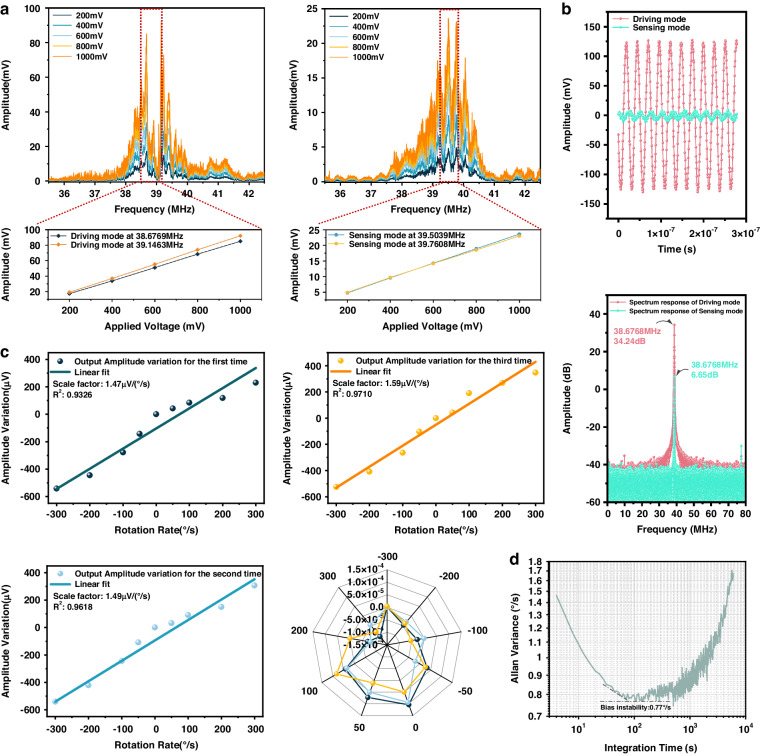
Performance of the toroidal SAW gyroscope **a** Using the open-loop test scheme, amplitude‒frequency curves with various input voltages in the driving mode and sensing mode can be obtained. **b** Time-domain signals and frequency-domain signals obtained by the gyroscope in the driving mode and sensing mode under a closed-loop test. **c** Rotational angular velocity—amplitude output curve of the SAW gyroscope and its fitting residuals. **d** Allan deviation of the SAW gyroscope

Using the open-loop test scheme, amplitude‒frequency curves with different input voltages can be obtained, as shown in Fig. 5a. With increasing input voltage, the amplitude‒frequency response curves in the driving mode and sensing mode exhibit an upward trend. The reason for this phenomenon is that the SAW amplitude generated based on the inverse piezoelectric effect is proportional to the alternating voltage applied to the FIDTs. It can also be seen that under the same input voltage, the amplitude in the driving mode is greater than that in the sensing mode, which is caused by the SAW reflectors in the driving direction. The reflector structure superimposes the traveling surface acoustic waves to generate a standing wave, which increases the amplitude of the SAW. As shown in the enlarged figure in Fig. 5a, the SAW amplitude in the driving mode with SAW reflectors is approximately 4 times the SAW amplitude in the sensing mode without reflectors. Figure 5b presents the characteristics of the SAW gyroscope during closed-loop testing. Under phase-locked control and amplitude-stabilized control, the gyroscope outputs time-domain signals in the driving mode and sensing mode. The measured data are further processed by fast Fourier transformation, and the results are shown in Fig. 5b. The driving mode vibrates at the resonant frequency of the gyroscopic stability, and the output signal in the sensing mode also exhibits the same vibration frequency.

When the gyroscope rotates at various rotation rates, the generated Coriolis force gradually changes. According to the working principle, the Coriolis force causes a secondary SAW, which can be obtained by FIDTs in the sensing direction. By using a multiplier to modulate the signal in the sensing direction, the relationship between the output amplitude of the SAW gyroscope and its rotational angular velocity is obtained, as shown in Fig. 5c. The figure shows that the sensitivities are 1.47 µV/(°/s), 1.49 µV/(°/s), and 1.59 µV/(°/s) within a range of ±300°/s in the three trials. The relative differences between these values may be caused by a systematic error of the installation and a random error of the sensor. The sensitivity of the gyroscope is approximately 1.51 µV/(°/s) on average, which is approximately one order of magnitude greater than that of the LIDT-structured SAW gyroscope. The fitting residuals of the three tests are also shown in Fig. 5c, and the results have a certain consistency. The Allan deviation of the SAW gyroscope is demonstrated in Fig. 5d. The output signal is recorded for 30 min with a sampling rate of 5 Hz during the static test, and the bias instability of the SAW gyroscope is 0.77°/s, which is an improvement of approximately one order of magnitude compared with that of the LIDT-structure SAW gyroscope.

## Discussion

In this paper, we propose a new strategy to improve sensor sensitivity by using FIDTs instead of LIDTs to increase the vibration velocity of SAW gyroscopes. However, many factors that affect the performance of the sensor should be considered in practical applications. Sensitivity degradation is an essential factor that cannot be ignored. Under harsh environments (high temperature, etc.) and external vibration, the thermal expansion of the piezoelectric crystal dramatically changes, which affects the material properties and SAW velocity, resulting in drift of the resonant frequency of the SAW resonator and ultimately significant degradation of the gyroscope sensitivity. Thus, a piezoelectric crystal with a low thermal expansion coefficient can be selected as the material of the SAW resonator to reduce the sensitivity of the resonant frequency to temperature and improve the temperature stability of the resonator. Moreover, hardware and software compensation can be applied to minimize the influence of temperature on the performance of the SAW sensor, such as the use of a differential structure, algorithmic compensation and on-chip micro-oven temperature control. Another important factor is that the Coriolis effect of the SAW gyroscope is very weak, resulting in a smaller output signal and severely affecting the practical use and performance of the sensor. Thus, in subsequent improved experimental tests, new detection methods need to be used to extract the weak signal of the sensor; for example, the advantages of high sensitivity, high stability and low noise in optical detection are helpful for accurate measurement by gyroscopes. The sensor performance can also be improved by optimizing the structural parameters of the SAW gyroscope or changing the substrate properties. Specifically, the number of pairs of FIDTs is proportional to the amplitude of the SAW gyroscope, and a thicker piezoelectric layer helps reduce energy loss and increase electromechanical coupling. By increasing the thickness of LiNbO_3_ and selecting a suitable number of FIDT pairs, the performance of the toroidal SAW gyroscope can be improved.

## Conclusion

In this paper, taking advantage of FIDTs, which can concentrate SAW energy to increase the vibration amplitude, a toroidal SAW gyroscope based on the standing wave mode is demonstrated and experimentally verified for the first time. The experimental results show that the sensitivity of the structure is 1.51 µV/(°/s), and the bias instability is 0.77°/s. Compared with those of the SAW gyroscope using LIDTs, the sensitivity and bias instability of the structure can be improved by an order of magnitude, which illustrates the superiority of the FIDT component. A comparison of the main specifications between our device and devices in the literature is shown in Table 2, which clearly shows that the gyroscope in this work has a relatively high performance. According to the theoretical analysis results, the mechanical sensitivity of the proposed structure has a certain dependence on its geometric parameters and the ambient temperature, so the sensitivity of the gyroscope can be further improved by optimizing the geometric parameters of the structure or implementing temperature compensation. The proposed SAW gyroscope adopts all-solid-state structures without any suspension-moveable elements and has great potential in harsh environments, such as geological exploration and oil drilling, in the future.

**Table 2:** Comparison of the performances of several reported SAW gyroscopes with our designed device

Reference	Working Mode	Die area (mm^2^)	Frequency (MHz)	Scale factor	Normalized scale factor	Bias instability
^25^	Progressive Wave Mode	1.4 × 0.6	80.2	52.35 Hz/deg/s	0.6527 × 10^−6^/deg/s	/
^47^	Progressive Wave Mode	9 × 9	98.6	0.431 Hz/deg/s	0.437 × 10^−8^/deg/s	/
^42^	Progressive Wave Mode	/	30	43 Hz/deg/s	1.4333 × 10^−6^/deg/s	/
^48^	Standing Wave Mode	10 × 10	74.2	0.0035 µV/deg/s	1.4 × 10^−9^/deg/s	/
^49^	Standing Wave Mode	20 × 20	115.5	0.025 µV/deg/s	0.5 × 10^−7^/deg/s	/
^50^	Standing Wave Mode	7.8 × 7.8	116.31	0.105 µV/deg/s	/	5.75°/s
This work	Standing Wave Mode	27.5 × 20	38.68	1.51 µV/deg/s	6.48 × 10^−5^/deg/s	0.77°/s

### Supplementary information

Supplementary Information for “A toroidal SAW gyroscope with focused IDT for sensitivity enhancement”
