# Supplementary material for: A toroidal SAW gyroscope with focused IDTs for sensitivity enhancement
Source: Microsyst Nanoeng. 2024 Mar 15;10:37. doi: 10.1038/s41378-024-00658-9 (PMC10940610; doi:10.1038/s41378-024-00658-9)
Supplement: Supplementary file 1 — Supplementary Information for “A toroidal SAW gyroscope with focused IDT for sensitivity enhancement” [file 41378_2024_658_MOESM1_ESM.docx]

**Supplementary Information for “A toroidal SAW gyroscope with focused IDTs for sensitivity enhancement”**

1. **The mechanical sensitivity of the SAW gyroscope**

When the alternating electrical voltage applied to the electrodes on both sides of the FIDTs, its cause particles on the piezoelectric medium to fluctuate through the inverse piezoelectric effect, forming SAW as shown in Fig. S1. The particle located at the metallic pillar on the piezoelectric medium is selected to effectively analyze SAW propagation characterize and Coriolis force by using elastic wave theory^1^.

In the piezoelectric material, due to the existence of the piezoelectric effect, the electromagnetic field equation and the acoustic field equation are not mutual independence but are coupled to each other through the piezoelectric equation. Therefore, not only the elastic wave equation but also the electromagnetic wave equation should be considered when the SAW is analyzed in the piezoelectric medium. The specific coupled wave equation is expressed as^2^:

$\left\{ \begin{matrix} \rho\frac{\partial^{2}u_{i}}{\partial t^{2}}-c_{ijkl}^{E}\frac{\partial^{2}u_{k}}{{\partial x}_{l}\partial x_{j}}-e_{kij}\frac{\partial^{2}\phi}{{\partial x}_{k}\partial x_{j}}=0 \\ e_{jkl}\frac{\partial^{2}u_{k}}{{\partial x}_{l}\partial x_{j}}-\varepsilon_{jk}\frac{\partial^{2}\phi}{{\partial x}_{k}\partial x_{j}}=0 \end{matrix} \right. （i,j,k,l=1,2,3）$ (S1)

where $\rho$ is the density of piezoelectric material; $c_{ijkl}^{E}$ is the elastic coefficients of the material when the electric field is fixed; $e_{kij}$ is the piezoelectric constant; $\varepsilon_{jk}$ is the permittivity; $\phi$ is the electric potential; and $u_{i}$ is the component of SAW displacement.

The elastic coefficients of the material affect the amplitude of SAW displacement, and its expression under the influence of temperature as follows^3,4^:

$c_{ijkl}^{'}=\frac{\sigma}{\xi-\xi_{0}}$ (S2)

where $\sigma$ is the stress vector; $\xi$ is the strain vector; and $\xi_{0}$ indicates the thermal induced strain.

The thermal expansion affected by temperature cause the SAW velocity various on the surface of substrate, and this velocity variation can be expressed as^5^:

$v_{SAW}^{'}=v_{SAW}(1-\alpha\Delta T)$ (S3)

where $\alpha$ is the temperature coefficient; $\Delta T$ is the temperature variation; and $v_{SAW}$ is the SAW velocity on the surface of substrate free from temperature.

Substitute Eq. (S2) and Eq. (S3) into the Eq. (S1), the SAW displacement can be given by:

$u_{i}=i_{0}^{'}\cos\left( \omega t-\frac{2\pi}{\lambda_{SAW}}X \right) （i=x,z）$ (S4)

where $u_{i}$ is the particle SAW displacement along the *i*th direction; $i_{0}^{'}$ is the amplitude affected by temperature along the *i*th direction; $\omega$ is the angular velocity, which can be expressed as $\omega={2\pi v_{SAW}^{'}}/{\lambda_{SAW}}={2\pi v_{SAW}(1-\alpha\Delta T)}/{\lambda_{SAW}}=\omega_{SAW}(1-\alpha\Delta T)$; $\omega_{SAW}$ is the angular velocity at the resonant frequency; $\lambda_{SAW}$ is the acoustic wavelength; $X$ is the displacement between the focal point and acoustic aperture, which can be expressed as $X={f_{L}W^{2}}/{\lambda_{SAW}\left( 1+\gamma\right)}$; $f_{L}$ is the geometric focal length; $W$ is the equivalent aperture of the central finger of FIDTs, which can be expressed as^6,7^ $W=2r\sin\left( {D_{a}}/2 \right)$; $r$ is the distance between the focal point and central finger; $D_{a}$ is the FIDT arc angle; and $\gamma$ is the anisotropy constant.

Surface acoustic waves propagate in the piezoelectric medium in an inverse elliptical trajectory based on the Rayleigh mode. Therefore, the particle vibration induced by SAW can be divided into the X-direction and the Z-direction, as shown in Fig.S1. When the gyroscope rotates along the X-axis, only the particle vibration along the Z-axis generates Coriolis force, so the SAW displacement along the Z-axis is considered in the subsequent analysis. The progressive SAWs generated by two FIDTs in the driving direction to form the standing wave mode, which displacement can be expressed as:

$U=2z_{0}^{'}\cos\left( \frac{2\pi}{\lambda_{SAW}}X \right)\cos\left( \omega t \right)$ (S5)

where $z_{0}^{'}$ the amplitude affected by temperature alone the z direction.

Based on Eq. (S5), the vibration of the metallic pillar located on the path of the standing wave can be expressed as:

$\nu_{p}=\dot{U}=-2z_{0}^{'}\omega\cos\left( \frac{2\pi}{\lambda_{SAW}}X \right)\sin\left( \omega t \right)$ (S6)

where $\nu_{p}$ is the vibration of the metallic pillar.

When the external rotational angular velocity $\Omega_{x}$ exists and the device rotates along the X axis, the metallic pillars generate Coriolis force $F_{c}$ along the Y axis, which can be expressed as follows:

$F_{c}=-2M_{p}\Omega_{x}\times\nu_{p}=4M_{p}\Omega_{x}z_{0}^{'}\omega\cos\left( \frac{2\pi}{\lambda_{SAW}}X \right)\sin\left( \omega t \right)$ (S7)

where $M_{p}$ is the total mass of the metallic pillars.

Based on the Eq. (S7), the Coriolis force alternate due to the waveform of the standing wave change over time. Under the piezoelectric effect, the alternating Coriolis force generate secondary SAW, which propagation direction perpendicular to the direction of standing wave. This secondary SAW is received by FIDTs in the sensing direction in the form of voltage, and the specific expression is as follows:

$U_{out}=\frac{dF_{cor}}{C_{e}}=U_{m}\sin\left( \omega t \right)$ (S8)

where $d$ is the piezoelectric constant; $C_{e}$ is the FIDTs capacitance, which is related to the number of pairs and equivalent aperture of FIDTs; $U_{m}$ is the output voltage amplitude, which can be expressed through simplification as $U_{m}={4M_{p}d\Omega_{x}z_{0}^{'}\omega}/{C_{e}}$ by considering that $\cos\left( {2\pi X}/{\lambda_{SAW}} \right)$ is approximately an integer multiple of $\cos\left( 2\pi\right)$.

Therefore, the mechanical sensitivity of the SAW gyroscope affected by temperature is expressed as the scale factor ($SF$), which can be obtained as follows:

$SF=\frac{U_{m}}{\Omega_{x}}=\frac{4M_{p}dz_{0}^{'}\omega_{SAW}(1-\alpha\Delta T)}{C_{e}}$ (S9)


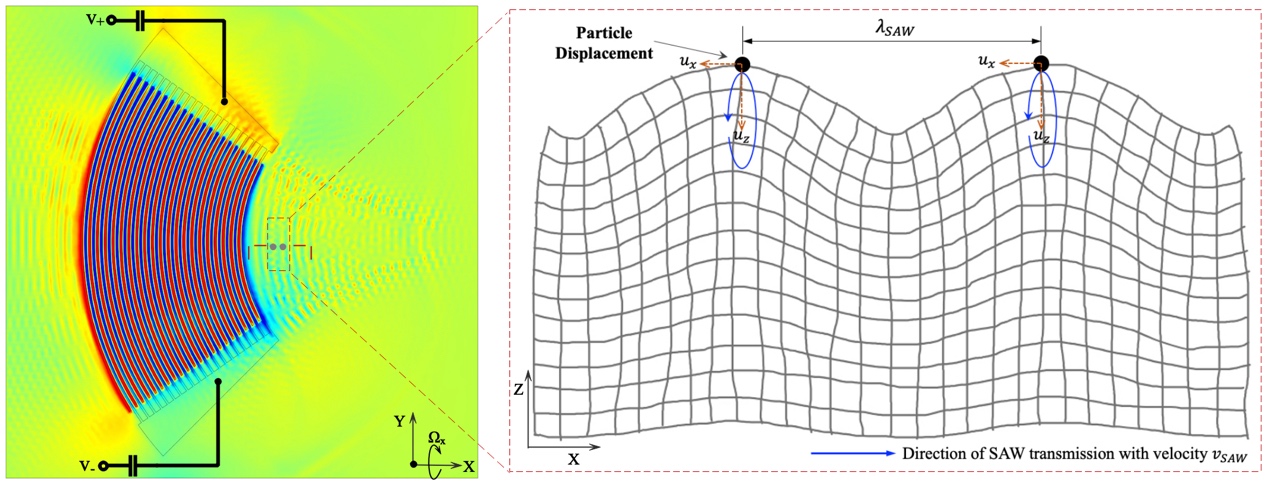


Figure S1. Simplistic representation of surface acoustic wave on surface piezoelectric substrate.

**References**

1. Wang, W., Shao, X., Liu, X., Liu, J. & He, S. Enhanced sensitivity of surface acoustic wave-based rate sensors incorporating metallic dot arrays. *Sensors* **14**, 3908–3920 (2014).

2. Lao, B. Y. Gyroscopic effect in surface acoustic waves. in *1980 Ultrasonics Symposium* 687–691 (IEEE, 1980).

3. Jiang, D., Xu, Y., Zhu, D. & Cao, Z. Temperature-dependent thermo-elastic parameter identification for composites using thermal modal data. *Adv. Mech. Eng.* **11**, 1687814019884165 (2019).

4. Haussühl, E. *et al.* Elastic properties of single crystal Bi12SiO20 as a function of pressure and temperature and acoustic attenuation effects in Bi12MO20 (M= Si, Ge and Ti). *Mater. Res. Express* **7**, 25701 (2020).

5. Zhang, S.-Y., Guo, L., Hu, A., Gao, Q.-S. & Lu, Z.-N. Temperature dependence of surface acoustic wave velocity in thin metal films. *Thin Solid Films* **202**, 171–179 (1991).

6. Kharusi, M. S. & Farnell, G. W. On diffraction and focusing in anisotropic crystals. *Proc. IEEE* **60**, 945–956 (1972).

7. O’Rorke, R., Winkler, A., Collins, D. & Ai, Y. Slowness curve surface acoustic wave transducers for optimized acoustic streaming. *RSC Adv.* **10**, 11582–11589 (2020).
